# Angina, a preoperative clinical sign for the development of delirium after transcatheter aortic valve implantation

**DOI:** 10.1111/ggi.14707

**Published:** 2023-10-31

**Authors:** Masashi Takeuchi, Hideaki Suzuki, Satoshi Miyata, Satoru Ebihara, Yasuda Satoshi

**Affiliations:** ^1^ Department of Cardiovascular Medicine Tohoku University Graduate School of Medicine Sendai Japan; ^2^ Department of Rehabilitation Medicine Tohoku University Graduate School of Medicine Sendai Japan; ^3^ Division of Brain Sciences Imperial College London London UK; ^4^ Teikyo University Graduate School of Public Health Tokyo Japan; ^5^ National Cerebral and Cardiovascular Center Suita Japan

Dear Editor,

Transcatheter aortic valve implantation (TAVI) is a well‐established treatment for aortic stenosis (AS) in older adults and/or patients with frailty, which leads to conventional symptoms, including angina and even death.^1^ Delirium after TAVI (DAT) occurs in up to 44% of patients and exacerbates long‐term prognosis and healthcare burden.^2^ Preoperative cerebral blood flow (CBF) in the insula, whose dysfunction is associated with delirium,^3^ is lower in patients with DAT relative to those without it.^4^ The insula is activated during visceral stimulation such as angina^5^ and plays an important role in pain processing.^6^ Chronic pain decreases CBF in regions associated with pain processing, including the insula.^7^ Pain is a potentially treatable cause of delirium.^8^ These evidence leads us to hypothesize that angina may decrease preoperative CBF in the insula, which increases the risk of DAT.

We studied 50 patients with New York Heart Association (NYHA) functional class II or III severe AS with TAVI,^4^ who underwent brain perfusion imaging preoperatively using ^99m^Tc‐ethyl cysteinate dimer single‐photon emission computed tomography (SPECT). AS‐related symptoms including angina were assessed by the local heart team who determined the indications and approaches for TAVI.^9^ The study protocol was approved by the Ethics Committee of the Tohoku University Graduate School of Medicine (no. 2023‐1‐250) and was conducted in compliance with the Declaration of Helsinki. Written informed consent was obtained from all the patients.

Brain perfusion SPECT image acquisitions and pre‐processing were performed as described previously.^4^ To test the association of angina with insular CBF, a whole brain voxel‐wise analysis was tested using SPM 12,^4^ with a model in which each voxel inside the insula of the pre‐processed perfusion map was a dependent variable and angina was an independent variable adjusted for age and sex. CBF values inside the regions exceeding a threshold of *P* = 0.05 with family‐wise error corrections for multiple comparisons in the voxel‐wise analysis^4^ were also calculated. Other statistical analyses were performed using Stata statistical software version 17 (StataCorp) at a significance threshold of *P* < 0.05. Continuous variables were expressed as mean ± standard deviation and were analyzed using the Student's *t*‐test. Nominal variables were analyzed using the Fisher's exact test. Structural equation modelling was used to explore the associations between angina, insular CBF, and DAT. Standardized correlation coefficients (β) and standard errors (SE) were shown.

Angina was reported in 12 patients (24%) prior to TAVI. Patients with angina had a higher prevalence of DAT relative to those without it (50% vs. 15.8%, *P* = 0.022), which was not a strong significance, while the other baseline characteristics were comparable between the two groups (angina vs. non‐angina: age, 84.8 ± 4.5 vs. 84.7 ± 0.7 years; female, 16.7% vs. 31.6%; aortic valve area index, 0.40 ± 0.03 vs. 0.42 ± 0.02 cm^2^/m^2^; left‐ventricular ejection fraction 61.6 ± 13.1 vs. 62.1 ± 12.6%; E/e′ 24.2 ± 11.5 vs. 19.0 ± 9.0; NYHA III, 91.7% vs. 60.5%; body mass index, 22.6 ± 0.8 vs. 22.8 ± 0.5; coronary artery disease, 50% vs. 26.3%; hypertension, 66.7% vs. 65.8%; diabetes, 41.7% vs. 15.8%; brain natriuretic peptide level, 484.5 ± 105.3 vs. 463.5 ± 116.0 pg/mL, all *P* > 0.05). No obvious cerebrovascular events occurred during the procedure. The whole‐brain voxel‐wise analysis showed angina‐associated regions inside the insula (Figure 1a), whose CBF was lower in patients with angina than those without it (53.8 ± 0.8 vs. 57.5 ± 0.4 mL/100 g/min, *P* < 0.001) (Figure 1b). Structural equation modeling showed that the positive association between angina and DAT was mediated by lower CBF in the insula (β = 0.216; SE = 0.095; *P* = 0.023), whereas no direct association was found between angina and DAT (β = 0.127; SE = 0.150; *P* = 0.398) (Figure 1c).

**Figure 1:**
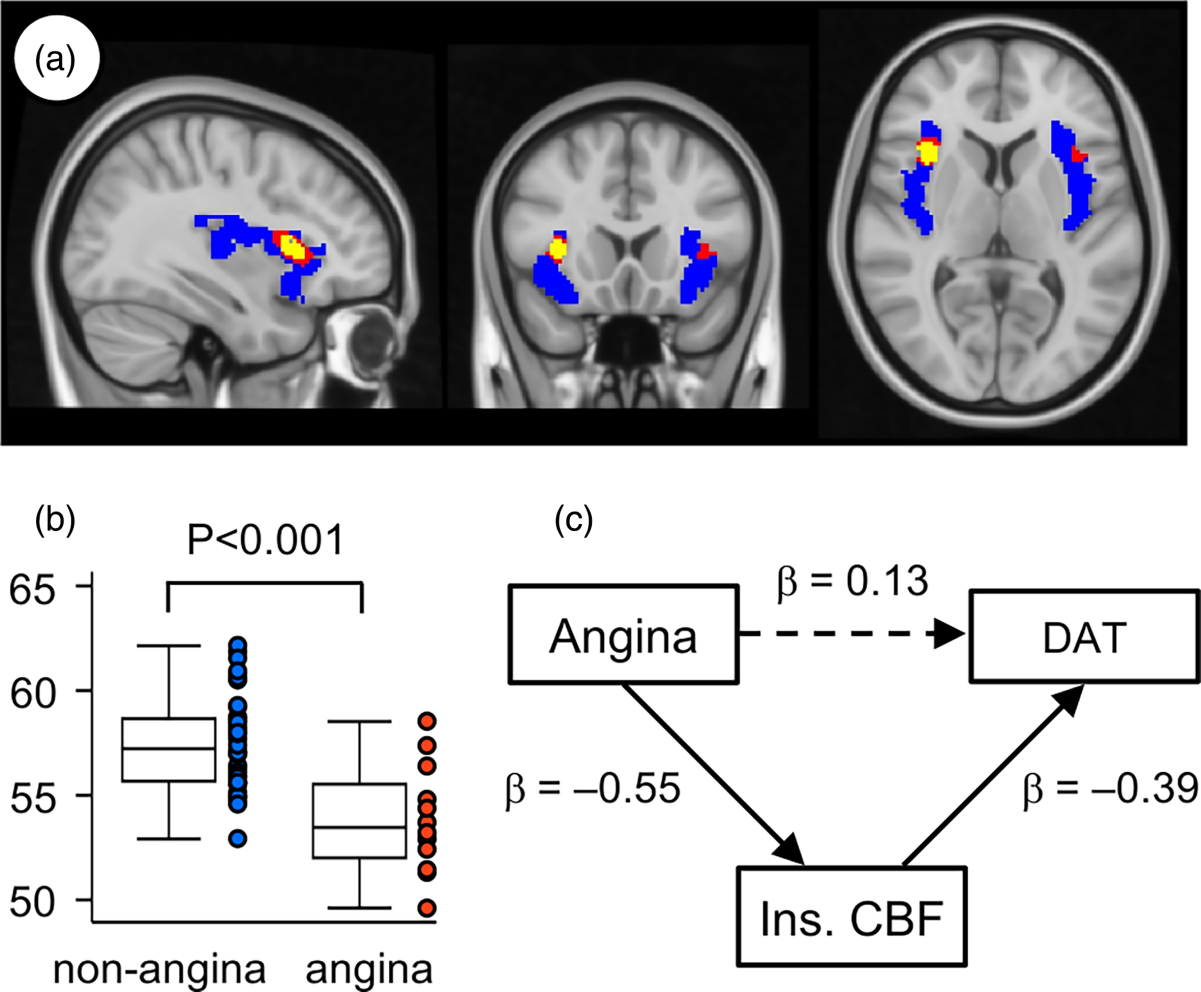
(a) Results of whole brain voxel‐wise analysis showing angina‐associated regions of the insula (yellow areas, *P* < 0.05 with family‐wise error corrections; red areas, *P* < 0.001, without corrections for multiple comparisons; blue areas, masks of the insula), in which the cerebral blood flow (CBF) was lower in the angina group (*N* = 12) compared with the non‐angina group (*N* = 38). (b) Box plots of CBF (mL/100 g/min) inside the regions exceeding the threshold of *P* < 0.05 with family‐wise error corrections. (c) Dots represent individual data. Structural equation model exploring associations between angina, CBF in the insula (Ins. CBF) and delirium after transcatheter aortic valve implantation (DAT). Standardized correlation coefficients are shown. Solid lines indicate statistically significant associations; dashed line indicates no statistically significant association. The model had a χ^2^ of 0.000, root mean square error of approximation less than 0.000, Akaike information criterion of 342, comparative fit index of 1.000, and Tucker–Lewis index of 1.000.

The present study demonstrated that preoperative CBF in the insula was lower in patients with AS with angina than in those without it. In addition, the positive association between angina and DAT was mediated by lower insular CBF. These findings indicate that angina may decrease the preoperative insular CBF, which increases the risk of DAT in patients with AS. There were no differences between the two groups in terms of potential factors that could induce angina, including aortic valve area index, NYHA class, coronary artery disease and brain natriuretic peptide level. Lower insular activity in the resting condition decreases the threshold of visceral sensation^10^ and might be associated with the perception of angina in the present study. Although insular CBF seems to be a good preoperative predictor of DAT,^4^ brain SPECT is not available in every hospital, is relatively expensive and causes exposure of patients to radiation. Angina may be a preoperative clinical sign and potentially a treatable cause of DAT, which is mediated by lower perfusion of the insula.

## Disclosure statement

The authors declare no conflict of interest.

## Data Availability

Data cannot be shared publicly because, based on the study protocol approved by the ethics committee of the Tohoku University Graduate School of Medicine (med‐kenkyo@grp.tohoku.ac.jp) (No. 2023‐1‐250), the participants in this study were given written informed consents, in which their data are not planned to be open to the public or to be shared with other researchers.
